# DFT-metadynamics insights on the origin of the oxygen evolution kinetics at the (100)-WSe_2_ surface

**DOI:** 10.1016/j.isci.2025.112045

**Published:** 2025-02-20

**Authors:** Fabrizio Creazzo, Kevin Sivula, Sandra Luber

**Affiliations:** 1 Department of Chemistry, University of Zurich, Zurich, Switzerland; 2 Institute of Chemical Sciences and Engineering, École Polytechnique Fédérale de Lausanne, Station 6, 1015 Lausanne, Switzerland

**Keywords:** Catalysis, Electrochemistry, Computational chemistry

## Abstract

Water oxidation or oxygen evolution reaction (OER) in electrochemical cells is considered to be a major bottleneck in the way of hydrogen production by electro-synthesis, mainly due to a sluggish kinetics that characterizes the OER steps. Layered transition metal dichalcogenides, such as WSe_2_, are emerging as promising non-precious electrocatalysts for water splitting due to their excellent activity and stability. This paper aims to shed light on the (100) WSe_2_-aqueous interface in catalyzing the slow kinetics of the OER in the context of water splitting electro-catalysis. We employ state-of-the-art DFT-metadynamics to explore reaction mechanisms, activation free energies, and catalytic sites. This study reveals an energetically preferred water-assisted OER mechanism, where proton transfer is facilitated by the surrounding aqueous environment. Our findings not only provide insights into the OER process but also offer a design strategy for optimizing WSe_2_-based catalysts and a modeling protocol for future DFT-based OER investigations.

## Highlights

• Oxygen evolution reaction through WSe_2_ catalyst via DFT-metadynamics
• Kinetics and reaction mechanisms of the oxygen evolution reaction
• An energetically preferred water-assisted oxygen evolution reaction mechanism

## Graphical abstract

**Figure:**
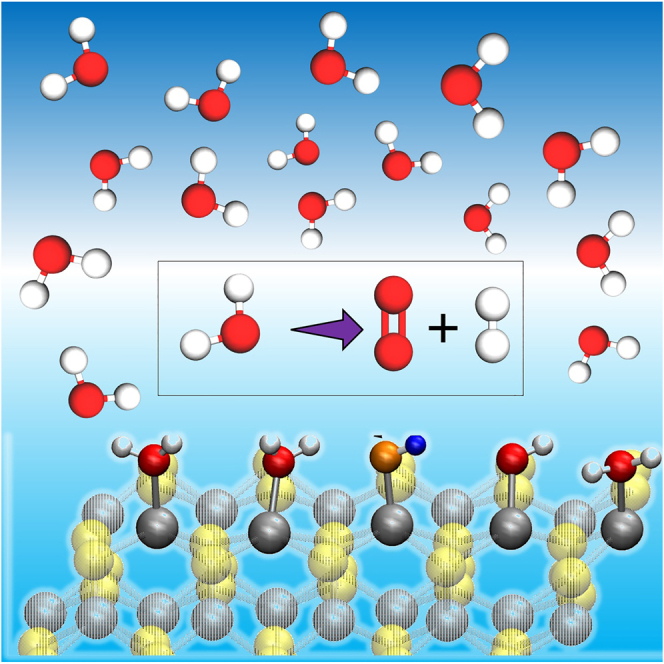

## Introduction

As earth abundant and stable materials, layered transition metal dichalcogenides (TMDCs), such as WSe_2_, have been gaining widespread attention for the design of improved electrocatalysts due to their unique chemical and physical properties. It has been shown that layered TMDCs can be exfoliated into two-dimensional sheets,^1^^,^^2^^,^^3^^,^^4^ similar to graphene. An example is the MoSe_2_ catalyst, which was first predicted by theory^5^^,^^6^ and then experimentally proven in several studies to have a better hydrogen evolution reaction (HER) activity than many non-precious metals.^7^^,^^8^^,^^9^ Similar experimental HER studies have been done on other TMDCs, including WS_2_, MoSe_2_, and WSe_2_,^10^^,^^11^^,^^12^^,^^13^^,^^14^ all of which showed promising activity for HER.

WSe_2_ is gaining a lot of interest among the family of TMDCs because of a larger size of W atoms compared to Mo counterparts, which can improve the catalytic performances of its specific layered-2D-structure.^15^^,^^16^ Moreover, the natural abundance of tungsten is slightly higher to that of molybdenum.^17^^,^^18^ All these makes WSe_2_ commercially more reliable for future large-scale applications among the TMDCs compounds.

One of the main research goals for improving WSe_2_ as catalyst has been to enhance catalytic performances^19^ focusing on the material morphology to expose more edge sites^20^^,^^21^ and obtaining doped structures. By this way, Huang et al. prepared a cobalt-doped WSe_2_ which exhibited an excellent electrocatalytic activity for the HER.^22^ Norskov et al., via density functional theory (DFT) static calculations, laid out that Se edges of WSe_2_ are active sites that enhance the electrochemical water reduction reaction to form H_2_.^23^ While the performance of TMDCs as water reduction electrocatalysts is now well understood, the prospect to employ TMDCs as OER electrocatalysts is much less established. Indeed, recently Ravishankar et al.^24^ reported an overpotential for the OER of 300 mV (10 mA cm^−2^) for a MoSe_2_@WSe_2_ composite electrocatalyst and good stability suggesting this class of materials could be also suitable for catalyzing the OER.

However, a comprehensive theoretical study of the structure and activity of WSe_2_ catalysts is currently lacking. While it is known that WSe_2_, and in general tungsten dichalcogenides, provide active sites for catalytic oxidation reactions,^25^^,^^26^ it is not established how this class of materials could have interesting activity into enhancing the OER. In this context, several open questions have to be addressed such as the involvement of these active sites in the OER, quantifying the available surface area of active sites, and the OER mechanism at these kinds of surfaces/interfaces.

The aim of the paper is to provide a deeper comprehension about the OER catalytic activity on WSe_2_ revealing mechanisms and kinetics behind the OER at atomistic details via DFT-based simulations. In previous studies,^27^^,^^28^^,^^29^^,^^30^^,^^31^ we have shown the critical role of an explicit bulk water environment on the reconstruction of the surface structures and how the dynamical behavior of liquid water placed at the interface is fundamental for a wide comprehension of interfacial reactions such as the OER. Most theoretical surface-science studies on WSe_2_^23^^,^^32^ are characterized by the lack of an explicit bulk water/solvent environment and its related dynamic behavior at the interface, due to simplifications behind the computational hydrogen electrode (CHE) approach (see details below). Few investigations employing the CHE approach include explicit solvation, such as in ref.^33^^,^^34^, but not for the WSe_2_ as catalyst. Such a paucity of information on the atomistic behavior of the local environment at the solid (WSe_2_)-liquid interface hampers the comprehension of fundamental chemical and physical phenomena such as the surface hydroxylation, the latter being a crucial step in the hydrogen and oxygen synthesis process. Accordingly, our current research aims to adopt state-of-the-art DFT-metadynamics simulations to reveal an understanding of (100)-WSe_2_-aqueous interface in catalyzing the OER in the context of electrocatalyzed water splitting. Metadynamics simulations go beyond static DFT by dynamically exploring reaction mechanisms, capturing rare events, and generating free energy profiles. This makes it a powerful tool for understanding the elementary steps of complex reactions, especially in concerted reactions where intermediates play a significant role^35^^,^^36^ which are challenging to capture with static DFT calculations. We will provide pieces of evidence on the critical kinetics that characterize the OER steps, revealing and comparing free-energy barriers behind the OER, in both gas and liquid phase, at the (100)-WSe_2_ catalyst surface. With the purpose of helping in the design of an efficient OER catalyst, our research is focused on understanding the role of an explicit solvent model and its dynamics in the kinetic evolution of oxygen. The paper is organized as follows: the section modeling of the OER and assumptions discusses about the modeling of the OER and selected assumptions made; the section *Our modeling of the OER at the (100)-W*Se_2_ describes the modeling approach used in this paper for our investigations of the OER in gas-phase and in explicit solvent model. The section OER at the hydrated (100)-WSe2 facet: gas-phase solvent model and section OER at the hydrated (100)-WSe2 facet: liquid-phase solvent model show and compare our OER metadynamics results in gas-phase and liquid-phase. Finally, in discussion section we summarize the main conclusions of our investigations. The adopted computational setup is described in section method details. Supplementary material can be found in supplemental information.

## Results

### Modeling of the OER and assumptions

One possibility to model the OER was proposed by Norskov et al.^37^^,^^38^ by following

(Equation 1) $$H_{2}O\left(l\right){+}^{*}\to HO^{*}+H^{+}+e^{-}$$

(Equation 2) $$HO^{*}\to O^{*}+H^{+}+e^{-}$$

(Equation 3) $$O^{*}+H_{2}O\left(l\right)\to HOO^{*}+H^{+}+e^{-}$$

(Equation 4) $$HOO^{*}\to O_{2}\left(g\right)+H^{+}+e^{-}$$

i.e., four electrochemical steps, each of which involves one $H^{+}/e^{-}$ transfer. The apex “^∗^” denotes an active surface site of the catalyst and $X^{*}$ a surface adsorbed X species. $HO^{*}$, $O^{*}$, and $HOO^{*}$ are denoted as OER intermediates, that is all adsorbed intermediates at the catalyst surface. O_2_ desorbs as molecular oxygen, creating a surface vacancy where a subsequent nucleophilic addition of another water molecule (Equation 4) can occur, and hence the OER can restart and continue. This OER model relies on the assumption of describing each reaction step as a proton coupled electron transfer (PCET)^39^ step. PCET reactions are therefore evaluated adopting the computational standard hydrogen electrode (CHE) based on gas phase $H_{2}$ formed from $H^{+}$ and $e^{-}$ as a reference reaction,^38^^,^^39^ allowing the comparison of theoretical electrochemical potentials with those measured in experiment. Due to its conceptual simplicity and the low computational cost in determining free energies of reaction intermediates, a wide adoption of the CHE approach describes most of static DFT-based studies.

However, the OER is significantly more difficult to model than assuming PCET steps together with the CHE approach. Furthermore, static-DFT investigations and the CHE approach are based on simplifications^40^ which are not necessarily justified in all cases, as highlighted in a recent review by Oberhofer.^41^ (1) The first simplification in most of other calculations is the lack of a direct (explicit) solvent and the omission of the latter in both modifying the surface catalyst (hydration/hydroxylation phenomena) and affecting DFT free energy calculations of reaction intermediates. Only few attempts with explicit solvation have been done,^33^^,^^34^ but not for the WSe_2_ as catalyst. (2) The assumption often employed in most of previous surface-science DFT calculations is the ideal pristine surfaces of catalysts. However, under reaction conditions, these tend to diverge from the clean, idealized surfaces and realistic catalysts are rarely pristine surface. This simplification makes challenging the identification of exact catalytic sites at the catalyst surface, giving a misleading or a partial view of the catalytic reaction phenomena at that ideal pristine surface. (3) Within the CHE approach, an additional simplification is that only the thermodynamic free energy differences between reactant, intermediates and products of a given reaction are estimated, assuming kinetic (free energy) barriers between these states as negligible. The activation barrier of an endergonic reaction step includes both a thermodynamic and a kinetic barrier. *Ab initio* thermodynamics can provide insights into electrochemical reaction kinetics if the activation barrier scales with thermodynamics (Brønsted-Polanyi-Evans [BEP] relation^42^^,^^43^^,^^44^) and the kinetic part is minimal. However, many electrocatalytic reactions such as the OER violate this implicit assumption, resulting in inconsistencies between *ab initio* thermodynamics and experimental data as well as *ab initio* kinetics.^45^ Free-energy barriers, as highlighted by ref.^41^, have no influence on the equilibrium constant of the reaction (the amount of reactants and products at the equilibrium), but they assess the dynamical properties of the reaction (such as the turnover frequency/rate) affecting therefore the minimum free-energy pathway and potentially leading to a different picture of the reaction chain. Recent years have seen an increase in detailed *ab initio* kinetics investigations in electrocatalysis,^46^^,^^47^^,^^48^ such as in previous studies^33^^,^^49^ where selected barriers (kinetic) have been estimated by employing nudged elastic band (NEB)^50^ and DFT-metadynamics computational techniques,^51^^,^^52^ mitigating the assumptions behind the CHE (and PCET) approach. First-principles kinetic studies showed that *ab initio* thermodynamics may not always accurately identify the critical step in complex electrocatalytic reactions. Although processing capacity has increased, *ab initio* kinetic studies remain computationally intensive. (4) Another drawback of the CHE approach is related to the potential-determining-step (PDS), on which the thermodynamic overpotential theory is based, that is not always identified as the rate-determining step (RDS).^53^^,^^54^ In electrocatalysis, the *ab initio* thermodynamic method is also utilized to derive kinetic conclusions by calculating the PDS and implicitly assuming that PDS = RDS and that thermodynamics governs the activation barriers. This crude approximation explains why mechanistic investigations based on the *ab initio* thermodynamics approach may not succeed, being PDS and RDS not connected to the same reaction step, affecting also the notion of the overpotential.

It is important to note that the thermodynamic overpotential is a thermodynamic measure for the activity and, as such, it is more in line with the PDS than the RDS which controls the (kinetic) reaction rate. This is a significant flaw in the approach since, as Koper discussed,^53^ the PDS and RDS do not always align. As a result, activity trends in a class of materials are not always accurately reproduced by the thermodynamic overpotential linked to the PDS. Conclusions drawn from the basic *ab initio* thermodynamics scheme of overpotential regarding activity and PDS need to be approached with caution.

Free-energy barriers are therefore crucial to better understand the exploitation of a given material as a catalyst. Ref.^41^ supports that the lack of free energy barrier estimation in previous studies is due to difficulties arising from performing constant potential (DFT) calculations^40^^,^^55^^,^^56^ and the computational cost associated with the dynamical calculations necessary to determine free energy profiles and thus barriers from first principles, i.e., the sampling of all environment degrees of freedom for each value of the reaction coordinate. This is what metadynamics techniques, and in general sampling techniques allow to map out at finite temperature.^57^^,^^58^ (5) Another challenging subject coming from the literature in the field is the identification of proper activity descriptors able to describe the suitability of a given compound as catalyst for a chosen reaction. Several activity descriptors have been adopted in previous works to define the OER activity, such as adsorption energies of intermediate species on catalyst surface,^37^^,^^38^ the number of d-electrons,^59^ the e_*g*_-band filling of transition-metal cations,^60^ the difference between the surface binding energies of $O^{*}$ and $HO^{*}$ reaction intermediates,^37^ the oxide formation energy,^61^ the accumulation of the magnetic moment^62^ and a convolution of individual overpotentials. More recently, the electrochemical-step symmetry index (ESSI)^54^ and electrochemical-step asymmetry index (ESAI)^63^ have been introduced as OER descriptors trying to unify potential and rate-determining steps by accounting for the applied potential.^64^ However, even the ideal value of 1.23 V has been challenged, leading to a subject of discussion.^54^^,^^63^ All of these highlight that there is currently no general consensus on the activity descriptors for catalysts^65^^,^^66^ and hence no unique agreement on how to calculate (theoretical) overpotential, and least of all on OER reaction mechanisms. Concerning the latter, it is usually assumed that the OER occurs at a single active site following the reaction mechanism (e.g., water nucleophilic attack) over the catalyst surface.^67^^,^^68^ However, there are pieces of evidence proposing a critical role played by the dynamic evolution of the catalyst/water interface in defining the reaction mechanisms and the related OER reaction barrier.^66^^,^^68^^,^^69^^,^^70^ For reactions calculated using DFT, the energy barrier of the RDS along the reaction coordinate is an explicit descriptor for the step’s kinetics. The choice of descriptors depends on the reaction mechanism, the material of interest, and the computational resources available. Combining these descriptors within a machine learning or data-driven approach has also become increasingly common for predicting catalytic activity and tuning catalysts for improved performance.^71^^,^^72^^,^^73^^,^^74^ Examples are also given by our previous works^75^^,^^76^^,^^77^ on predicting descriptors for a bunch of reactions (Diels–Alder Cycloaddition, Keto–Enol Tautomerism, water oxidation, *etc.*), including liquid-phase solvent model, by using an unsupervised machine learning technique.

### Our modeling of the OER at the (100)-WSe_2_ surface at the interface with water

**Figure 1:**
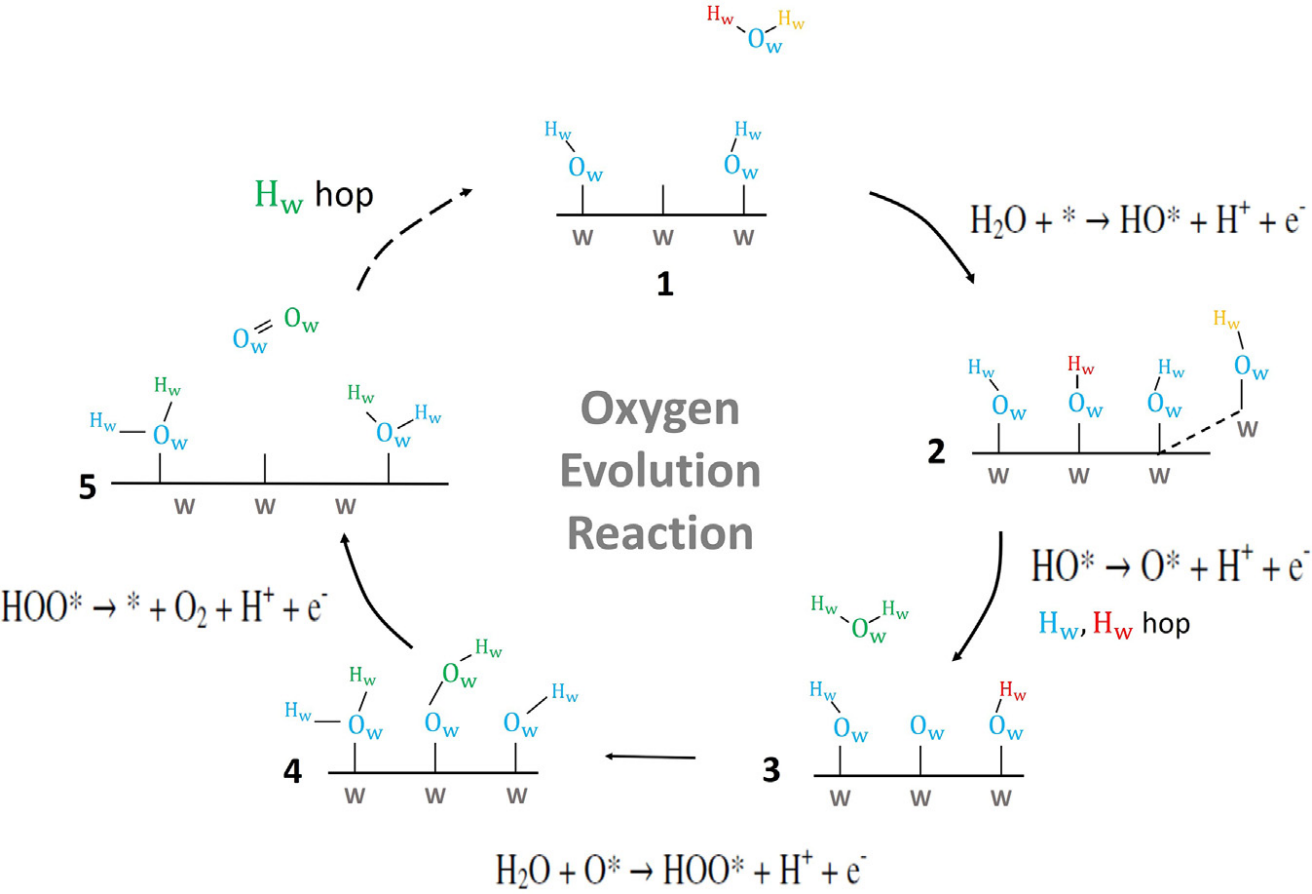
Proposed mechanism of the OER cycle as our reference pathway taking place via a water attack and surface adsorbed intermediates $HO^{*}$, $O^{*}$ and $HOO^{*}$ at the (100)-WSe_2_ surface

The modeling of heterogeneous catalytic systems’ reactivity is nontrivial and fraught with difficulties. Especially for systems with well-defined catalytic sites and at low temperatures and coverage, *ab initio* static simulations yield results that could facilitate the interpretation of experimental data. However, these circumstances are frequently far from catalytic processes in practical experimental settings that require considerably more complexity than static DFT investigations and CHE approaches. In our DFT-metadynamics calculations, no CHE is included explicitly or implicitly as done e.g., in previous static calculations.^33^^,^^39^^,^^78^^,^^79^ We follow the OER pathway (Equations 1, 2, 3, and 4) as suggested in ref.^37^^,^^38^. However, with the aim to go beyond standard static calculations and to mitigate some of the abovementioned simplifications, we will adopt DFT-well tempered (WT) metadynamics including an explicit bulk water environment and a finite temperature to reveal the OER mechanism in a framework where catalyst (not-pristine but hydrated/hydroxylated, see section method details for details) is always in direct contact with an explicit liquid environment, trying to be closer to the experimental setup. The OER mechanism is evaluated by assuming the free-energy barrier as a kinetic descriptor, allowing the identification of the minimum energy pathway and the RDS (not assuming that PDS = RDS as in the CHE approach). Our investigation is also extended to gas-phase OER in order to compare this latter in gas and liquid phase. The employed DFT-WT metadynamics technique will allow to assess dynamical properties of the specific OER mechanism by estimating the free-energy barrier for each OER step at the (100)-WSe_2_ catalyst surface with the inclusion of an explicit bulk water environment. The reference OER pathway we adopt for our metadynamics calculations at the (100)-WSe_2_ surface is schematically shown in Figure 1, for which the reaction mechanism involves the oxidation of a water molecule via a nucleophilic water attack to the (100)-WSe_2_ catalyst surface. (100)-WSe_2_ catalyst acts as anode *reservoir* of electrons, these latter flow toward the counter-cathode (not included in the calculations in this work) during the evolution of oxygen cycle. No external biases such as external potential or pH changes have been adopted. Moreover, protons/electrons are removed from the simulation box after each PCET step. Previous studies suggested that PCET steps are concerted rather than decoupled and that the reaction intermediates refer to the OH, O, and OOH adsorbates,^38^ supported by the spectroscopic identification of the OOH adsorbate.^80^^,^^81^ We can therefore consider the proton-electron transfers (toward the catalyst surface and/or water) as coupled phenomena, making unnecessary to separately identify the chemical potential of the proton and electron. This approach has been successfully adopted in previous kinetic OER studies.^47^^,^^82^

**Figure 2:**
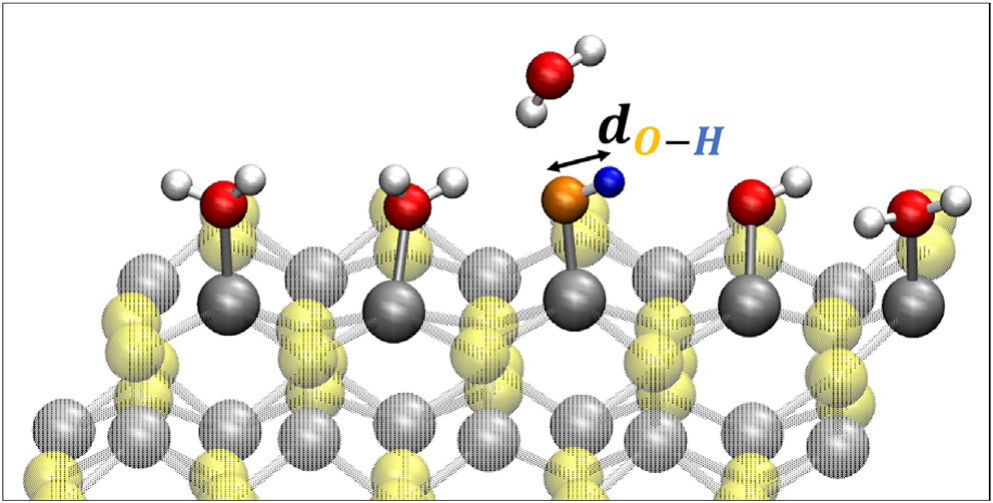
Modeling of reaction step 2 → 3 for our WT metadynamics investigations at the (100)-WSe_2_ surface The black arrow highlights the chosen reaction coordinate.

The reaction step 1 → 2 (i.e., $H_{2}O\left(l\right){+}^{*}\to HO^{*}+H^{+}+e^{-}$) has been found (energetically) spontaneous occuring during our (unbiased) DFT-MD simulations with the dissociation of a water molecule and the consequent formation of surface adsorbed $OH^{*}$ and $H^{*}$ at (100)-WSe_2_ facet, see details in our previous paper.^31^ Accordingly our WT metadynamics investigations have been focused on reaction steps 2 → 3 ($HO^{*}\to O^{*}+H^{+}+e^{-}$), 3 → 4 ($O^{*}+H_{2}O\left(l\right)\to HOO^{*}+H^{+}+e^{-}$) and 4 → 5 ($HOO^{*}\to O_{2}\left(g\right)+H^{+}+e^{-}$) as in the following. Reaction step 2 → 3 ($HO^{*}\to O^{*}+H^{+}+e^{-}$): surface adsorbed $OH^{*}$ is dissociated in order to create an available $O^{*}$ at the (100)-WSe_2_ surface. The distance between the oxygen $O^{*}$ and the H is chosen as unique reaction coordinate (i.e., collective variable for metadynamics) as displayed in Figure 2.

**Figure 3:**
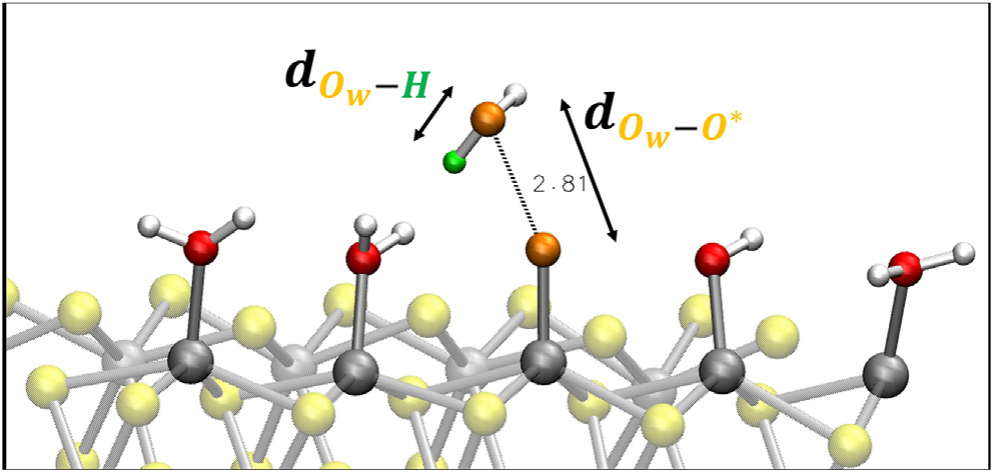
Modeling of reaction step 3 → 4 for our WT metadynamics investigations at the (100)-WSe_2_ surface Black arrows highlight chosen reaction coordinates. See text for details.

Reaction step 3 → 4 ($O^{*}+H_{2}O\left(l\right)\to HOO^{*}+H^{+}+e^{-}$): formation of surface adsorbed intermediates $HOO^{*}$. Two reaction coordinates are adopted: (1) distance between the oxygen $O_{w}$ (of the reactant water molecule) and $O^{*}$ that is surface-adsorbed oxygen atom (see Figure 3) and (2) distance between the oxygen $O_{w}$ and one hydrogen *H* of the same water molecule (see Figure 3).

**Figure 4:**
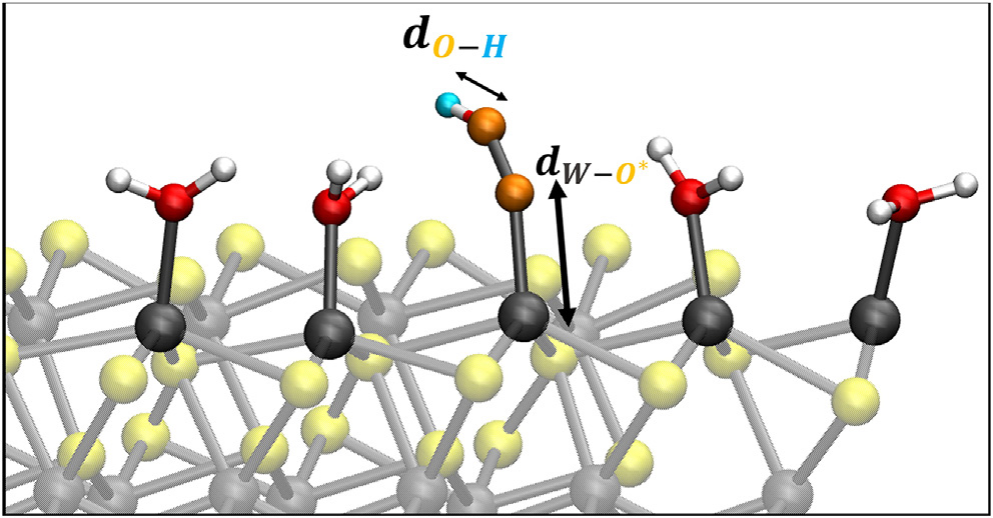
Modeling of reaction step 4 → 5 for our WT metadynamics investigations at the (100)-WSe_2_ surface Black arrows highlight chosen reaction coordinates. See text for details.

It has been shown that using the simple $O^{*}$-$O_{w}$ distance as a reaction coordinate can provide a misleading reaction pathway (and hence free-energies) because relevant features of the process of interest can be neglected.^51^ The formation of the O−O bond presumes a deprotonation of the nucleophile (water) ‘at the same time’. Proton acceptors near the active site facilitate the O-O bond formation.^47^^,^^82^^,^^83^ In this reaction step, being typically the energetically most demanding, a careful analysis has to be done without the risk of neglecting relevant features. Accordingly, we decided here to consider two reaction coordinates (not only one) in order to explore the phase space related to both deprotonation of water and $HOO^{*}$ intermediate formation. Reaction step 4 → 5 ($HOO^{*}\to O_{2}\left(g\right)+H^{+}+e^{-}$): deprotonation of $HOO^{*}$ and the consequent $OO^{*}$ desorption from the (100)-WSe_2_ surface. The chosen reaction coordinates are as follows: (1) distance between *O* and *H* within the $HOO^{*}$ for the deprotonation of $HOO^{*}$ (see Figure 4) and (2) distance between the oxygen $O^{*}$ and the *W* surface atom for the $OO^{*}$ desorption (see Figure 4).

Once $O_{2}$ desorbs from the surface, an additional water molecule can be adsorbed/dissociated at the surface to have again surface $O^{*}$ (or $OH^{*}$), assuring the OER cycle can restart and continue. This nucleophilic addition of a further water molecule has been proved to occur spontaneously (i.e., water molecule addition in step 1 → 2) and hence is not investigated in our OER metadynamics.^47^^,^^68^^,^^82^^,^^84^^,^^85^ The same is valid for spontaneous proton hopping events that occur between neighboring $O^{*}$ (or $OH^{*}$) surface sites (step 5 → 1). The reaction step with the largest free energy barrier is assumed as the RDS among reaction steps 2 → 3, 3 → 4 or 4 → 5. It should be mentioned that, several theoretical and experimental studies about heterogeneous catalysis^47^^,^^68^^,^^82^^,^^84^^,^^85^^,^^86^ have shown that if only one surface active site is available for the OER, the $O-O^{*}$ bond formation is hard to achieve due to the repulsion between the electron-rich surface O and the O of the water molecule. The high localization of the electronic charge can cause a high-energy barrier for the $O-O^{*}$ bond formation.^47^^,^^82^ Instead, the cooperation of two adjacent surface active sites provide an energetically easier water dissociation at the catalyst surface to form the desired $O-O^{*}$ surface adsorbed species. For these highlighted reasons, and for the fact that several chemical species are possible OER catalyst exposed sites – such as $\mu_{1}$-OH_2_, $\mu_{1}$-OH and $W_{cus}$ atoms at the hydrated/hydroxylated (100)-WSe_2_ surface, - all metadynamics calculations (in gas and liquid phase) reported here for the OER investigations are performed with the assumption that, at least, two adjacent $\mu_{1}$-OH (i.e., W−OH) surface sites are available at the surface catalyst, as also outlined in the proposed reference OER mechanism in Figure 1. Our OER metadynamics investigations will be done, first, at the hydrated/hydroxylated (100)-WSe_2_ surface at the interface with only one explicit water molecule (at a distance of around 3 Å from the catalyst’ surface), and second, by including an explicit slab of 120 water molecules at the interface (with a distance of around 3 Å between the catalyst’ surface and the first water layer at the interface), thus allowing a consistent comparison of our results and a better comprehension of how a (full explicit) liquid water environment can affect the OER kinetics and the catalytic properties of the (100)-WSe_2_ surface.

### OER at the hydrated (100)-WSe_2_ facet: Gas-phase solvent model

**Figure 5:**
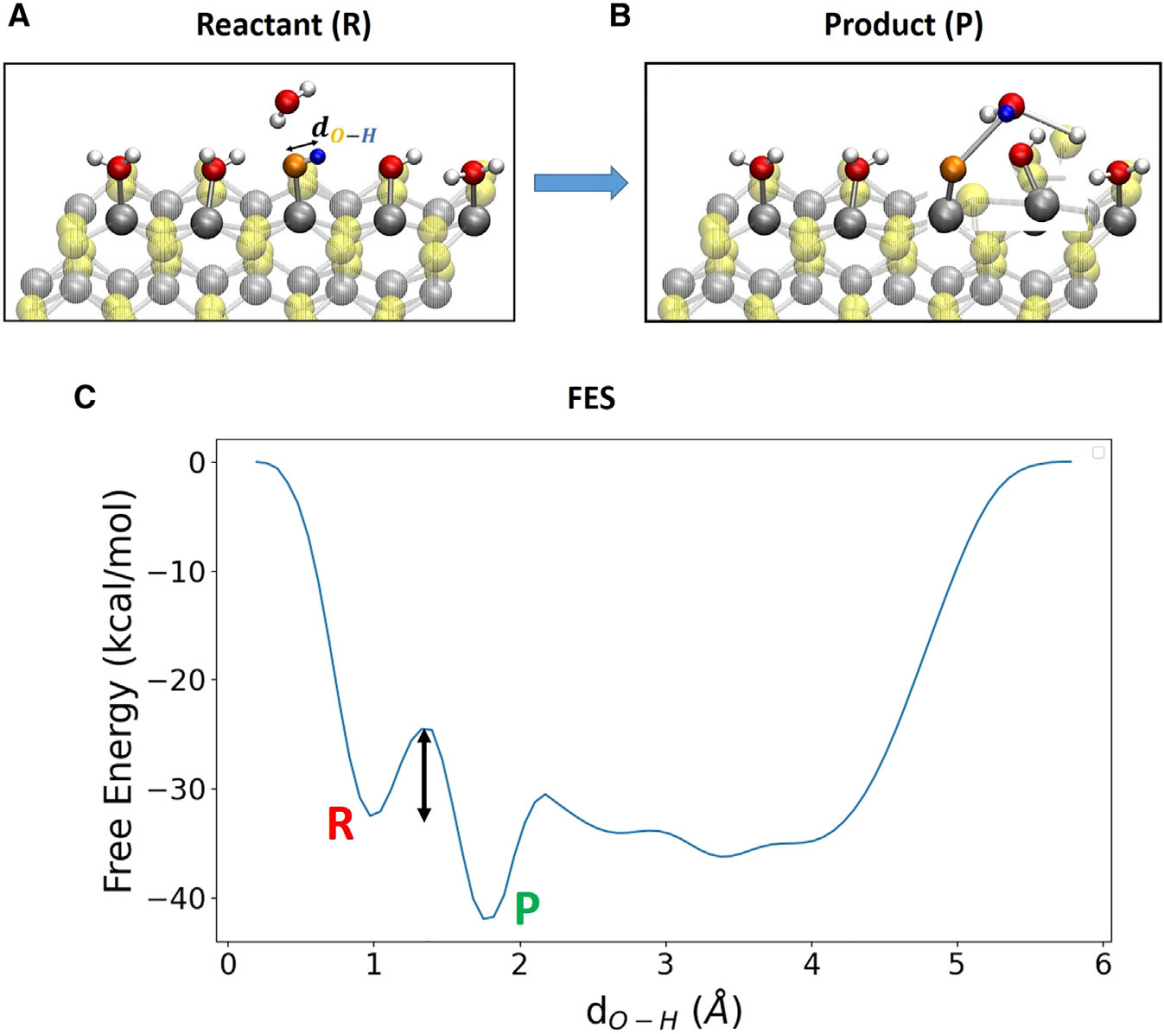
Free energy profile Snapshots from (DFT-MD) WT metadynamics simulations of reactant (A) and product (B) states for the gas-phase step 2 → 3 at the (100)-WSe_2_ facet. The black arrow highlights the chosen reaction coordinate. The oxygen atom in orange color and the proton in blue color highlight the chosen reactant atoms. (C) The associated free-energy profile. R and P denote the reactant and product state, respectively. The free-energy scale in the y axis is in kcal/mol. The reaction coordinate is expressed as distance (Å) in the x axis. The black arrow highlights the free-energy barrier.

As highlighted in the previous section, the OER reaction step 1 → 2 (i.e., $H_{2}O\left(l\right){+}^{*}\to HO^{*}+H^{+}+e^{-}$) has been found to be a (free-energy) barrierless step, with the dissociation of a water molecule and the consequent formation of surface adsorbed $OH^{*}$ and $H^{*}$ at (100)-WSe_2_ facet.^31^ OER reaction steps 2 → 3, 3 → 4, and 4 → 5 are investigated at the (100)-WSe_2_ surface in gas-phase where only one explicit interfacial water molecule is present. With the aim of exploring the OER at the (100)-WSe_2_ surface, the free-energy barrier for each reaction step is therefore estimated and discussed below. Reaction step 2 → 3 ($HO^{*}\to O^{*}+H^{+}+e^{-}$): surface adsorbed $OH^{*}$ is dissociated in order to create an available $O^{*}$ at the surface. The dissociated proton from $OH^{*}$ goes toward the water molecule and, at the same time, a proton from this latter hops toward a surface Se atom (through a simultaneous H_3_O^+^ formation/proton hop), as shown in Figures 5A and 5B. The gas-phase free energy profile by means of WT metadynamics for the reaction step 2 → 3 is shown in Figure 5C.

**Figure 6:**
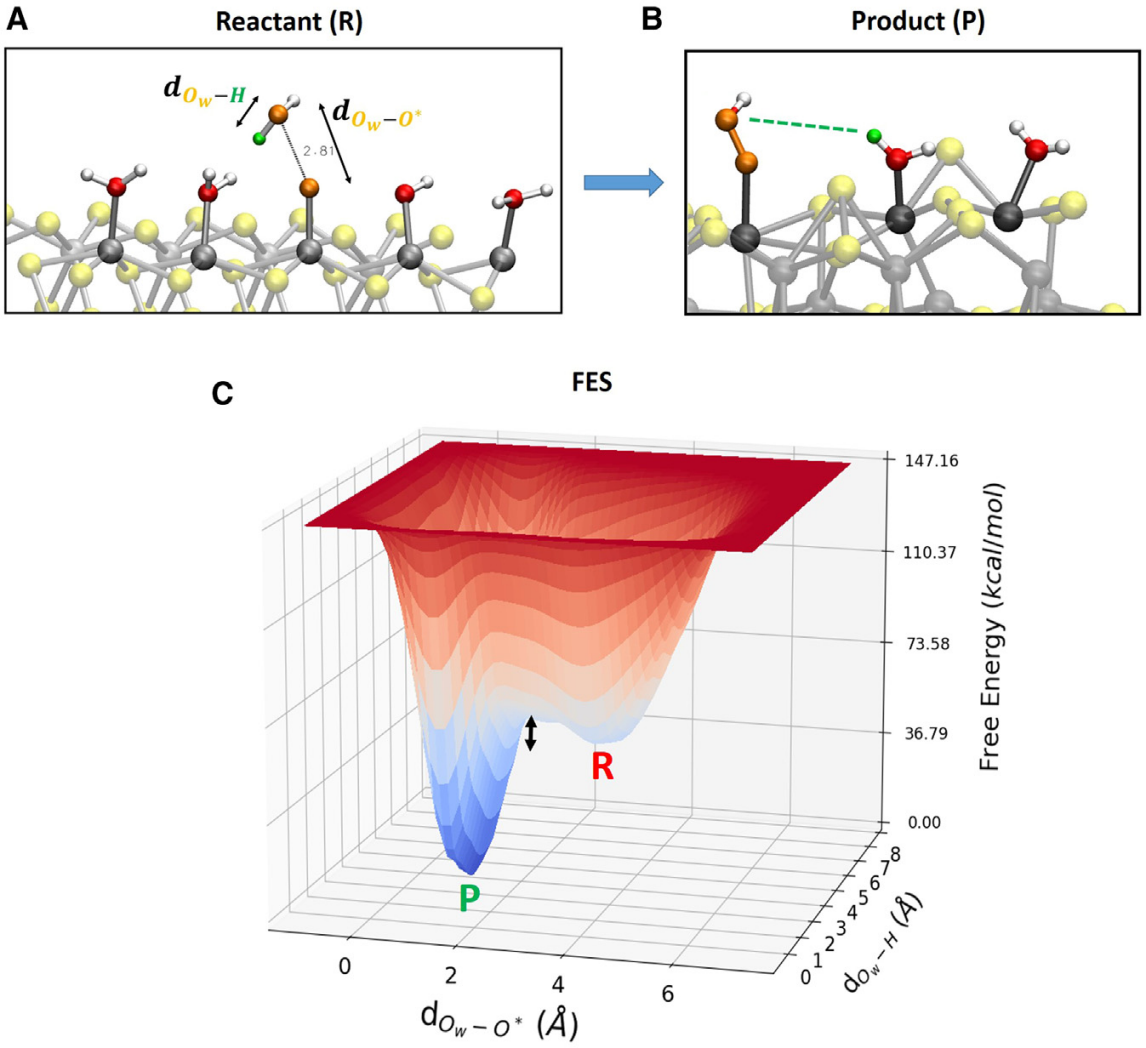
Free energy surface Snapshots from (DFT-MD) WT metadynamics simulations of reactant (A) and product (B) states for the gas-phase step 3 → 4 at the (100)-WSe_2_ facet. Black arrows highlight chosen reaction coordinates. (C) The associated 3D free-energy surface. R and P denote the reactant and product state, respectively. The free-energy scale in the z axis is in kcal/mol. Reaction coordinates are expressed as a distance (Å) in x- and y axis. The black arrow highlights the free-energy barrier.

The free-energy barrier is ∼ 9 kcal/mol (∼0.4 eV). The saddle-point at around 3 Å is due to (statistical) free-energy fluctuations given by the water molecule that during its dynamics proceeds to sample different points of the reaction coordinate. The dynamics of the local environment could play a role in defining saddle-points during metadynamics sampling, as outlined in ref.^87^. The presence of this saddle-point does not change our results/conclusions. Reaction step 3 → 4 ($O^{*}+H_{2}O\left(l\right)\to HOO^{*}+H^{+}+e^{-}$): starting water molecule position has been chosen as the water molecule above (closest to) the selected surface oxygen atoms $O^{*}$ in orange color in Figure 6. The free energy surface is depicted in Figure 6C.

**Figure 7:**
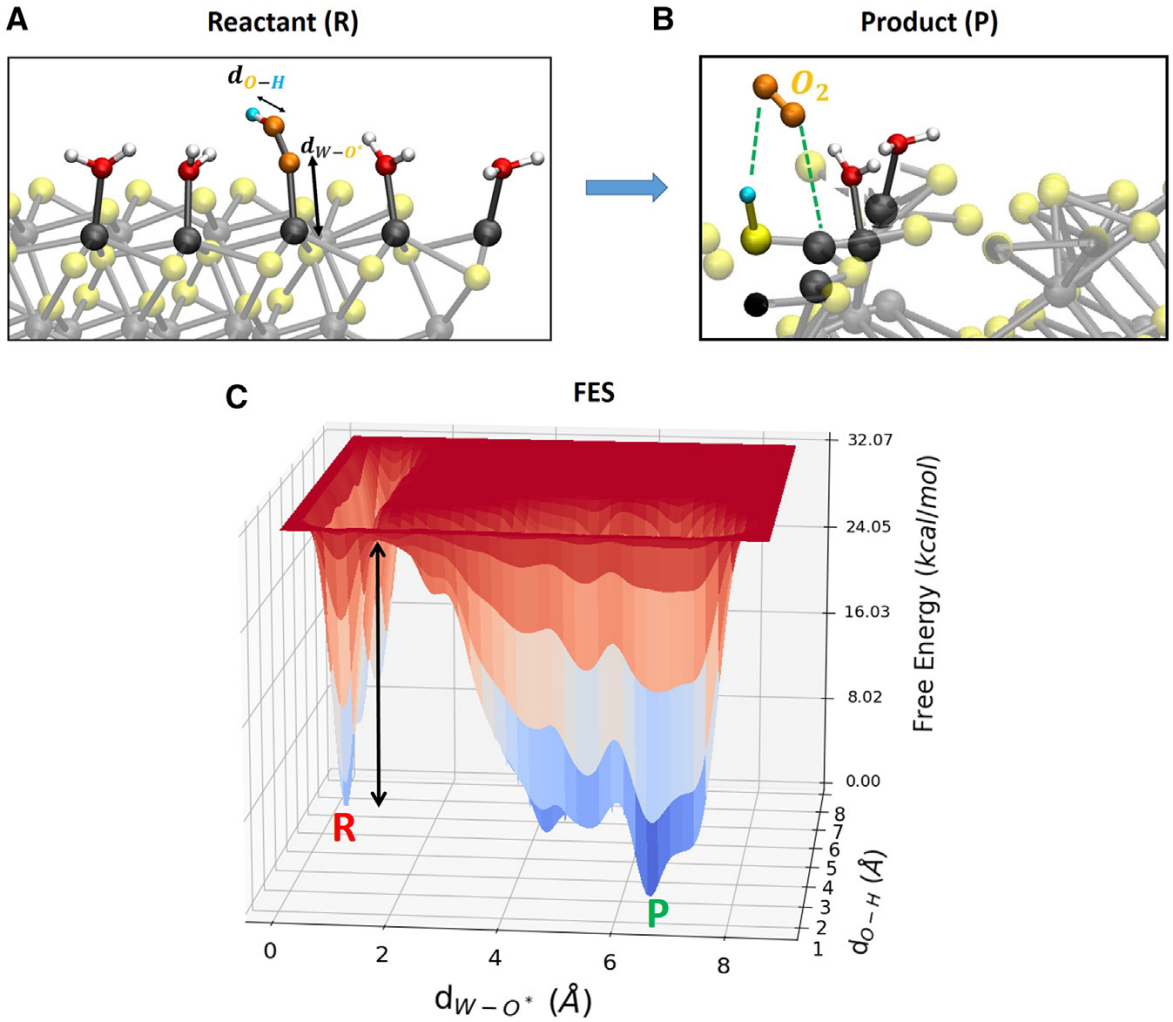
Free energy surface Snapshots from (DFT-MD) WT metadynamics simulations of reactant (A) and product (B) states for the gas-phase step 4 → 5 at the (100)-WSe_2_ facet. Black arrows highlight chosen reaction coordinates. (C) The associated 3D free-energy surface. R and P denote the reactant and product state, respectively. The free-energy scale in the z axis is in kcal/mol. Reaction coordinates are expressed as a distance (Å) in x- and y axis. The black arrow highlights the free-energy barrier.

Reaction step 3 → 4, that is the dissociation of the reactant water molecule and consequently, the formation of surface adsorbed intermediates $HOO^{*}$ (formation of $\mu_{1}$-OOH surface termination), as depicted in Figures 6A and 6B. The dissociated proton (from the water molecule) hops toward a neighbor $HO^{*}$ surface termination, see Figure 6B. The free-energy barrier is ∼ 15 kcal/mol (∼0.7 eV). Reaction step 4 → 5 ($HOO^{*}\to O_{2}\left(g\right)+H^{+}+e^{-}$): the deprotonation of $HOO^{*}$ and consequently the $OO^{*}$ desorption from the (100)-WSe_2_ surface. The dissociated proton is surface adsorbed by a neighbor Se atoms, see Figure 7B. The free-energy barrier is ∼ 23 kcal/mol (∼1.0 eV).

Among the investigated reactions, the reaction step with the largest free-energy barrier has been identified in the concerted reaction step 4 → 5 where ∼ 23 kcal/mol (∼1.0 eV) has to be overcomed for the OER to occur in gas-phase. The concerted reaction step 4 → 5 is therefore identified as the RDS of the gas-phase OER at the (100)-WSe_2_ facet. The O_2_ (gas) product has been found in its electronic ground state, namely the triplet state, with two unpaired electrons in the orbital level n=2 (*n* is the principal (orbital) quantum number). The same will be valid also for the O_2_ obtained in our OER liquid phase investigation in the next section OER at the hydrated (100)-WSe2 facet: liquid-phase solvent model. These findings are in agreement with a previous study.^88^ Gas-phase OER transition states (TSs) are displayed in Figures S2 in the supplemental information.

### OER at the hydrated (100)-WSe_2_ facet: Liquid-phase solvent model

**Figure 8:**
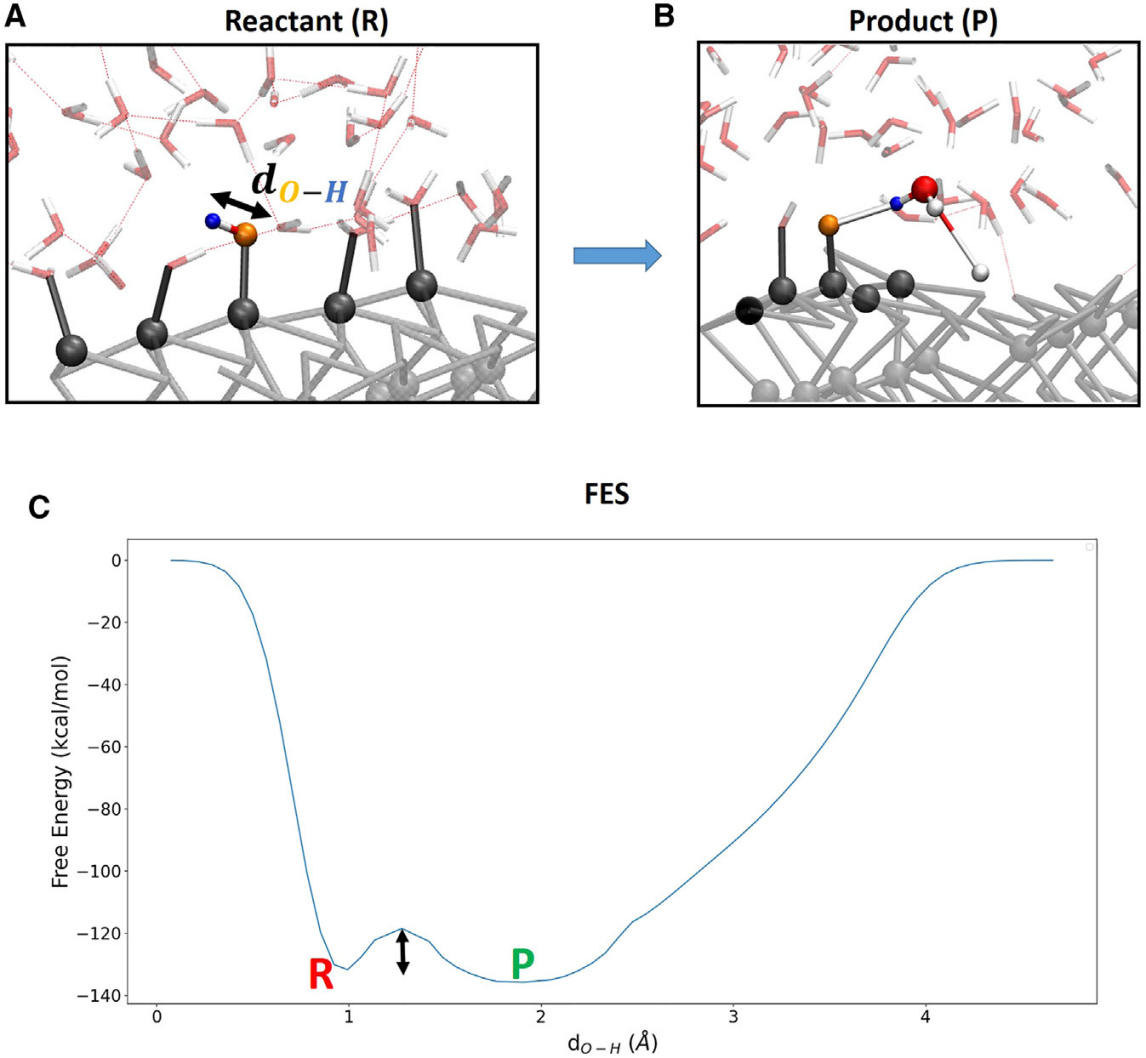
Free energy profile Snapshots from (DFT-MD) WT metadynamics simulations of reactant (A) and product (B) states for the liquid-phase OER step 2 → 3 at the (100)-WSe_2_ facet. The black arrow highlights the chosen reaction coordinate. (C) The associated free-energy profile. R and P denote the reactant and product state, respectively. The free-energy scale in the y axis is in kcal/mol. The reaction coordinate is expressed as a distance (Å) in the x axis. The black arrow highlights the free-energy barrier.

Following our purpose to investigate the OER close to actual experimental conditions, we now take into account a water slab (120 water molecules, density ∼ 1.0 g/cm^3^) at the interface with the hydrated (100)-WSe_2_ to perform our OER investigation. In this way, we aim to overcome the aforementioned limit of the current literature by adopting implicit solvent approaches, or at the best, only one water molecule or a water monolayer in contact with the catalyst surface that does not match with experimental conditions. As already done for the OER gas-phase model in the previous section, the free-energy barrier is therefore estimated for each OER step shown in Figures 2, 3, and 4 by means of WT-metadynamics. For the purpose of comparison, starting configurations (reactant species) are chosen as the same seen in gas-phase, but now considering a surrounding explicit water environment. As partly expected, we will see in the following that the presence of the interfacial aqueous environment now leads to an OER minimum energy pathway that is different from the one seen in the gas-phase, characterized by a noticeable lower free-energy barrier. Reaction step 2 → 3 ($HO^{*}\to O^{*}+H^{+}+e^{-}$): surface adsorbed $OH^{*}$ is dissociated in order to create an available $O^{*}$ at the surface. The dissociated proton from $OH^{*}$ goes toward a neighbor water molecule (belonging to the explicit water environment) and, at the same time, a proton from this latter hops toward a surface atom (through a simultaneous H_3_O^+^ formation/proton hop), as shown in Figures 8A and 8B. The free energy profile by means of WT metadynamics for the reaction step 2 → 3 is shown in Figure 8C.

**Figure 9:**
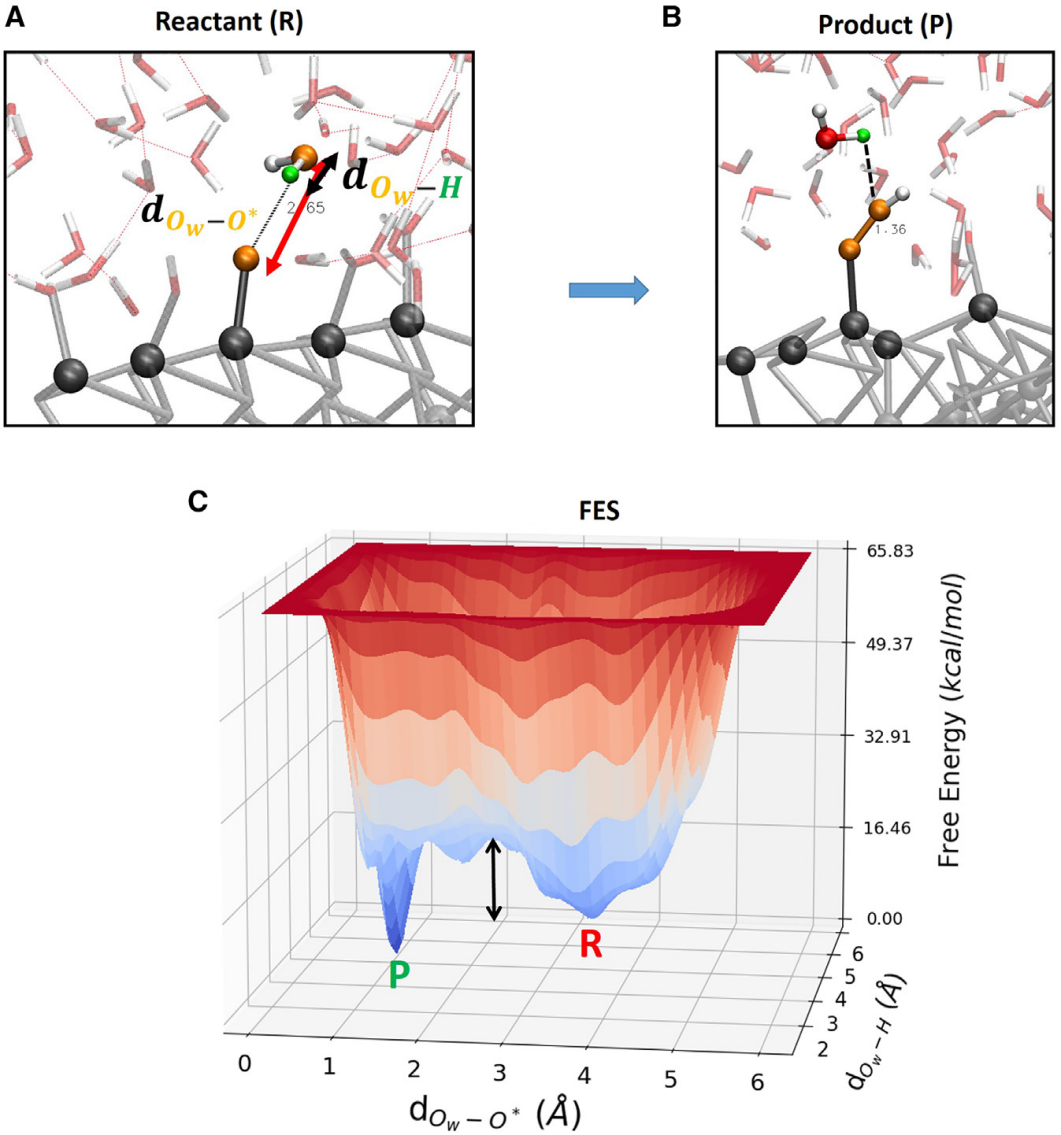
Free energy surface Snapshots from (DFT-MD) WT metadynamics simulations of reactant (A) and product (B) states for the liquid-phase OER step 3 → 4 at the (100)-WSe_2_ facet. Black and red arrows highlight chosen reaction coordinates. The dissociated proton (in green color) hops toward a nearby water molecule. (C) The associated 3D free-energy surface. R and P denote the reactant and product state, respectively. The free-energy scale in the z axis is in kcal/mol. Reaction coordinates are expressed as a distance (Å) in x- and y axis. The black arrow highlights the free-energy barrier.

The free-energy barrier is ∼ 8 kcal/mol (∼0.4 eV), a value quite comparable with the one found in gas-phase of ∼9 kcal/mol for the same step. Reaction step 3 → 4 ($O^{*}+H_{2}O\left(l\right)\to HOO^{*}+H^{+}+e^{-}$): the free energy surface is depicted in Figure 9C.

**Figure 10:**
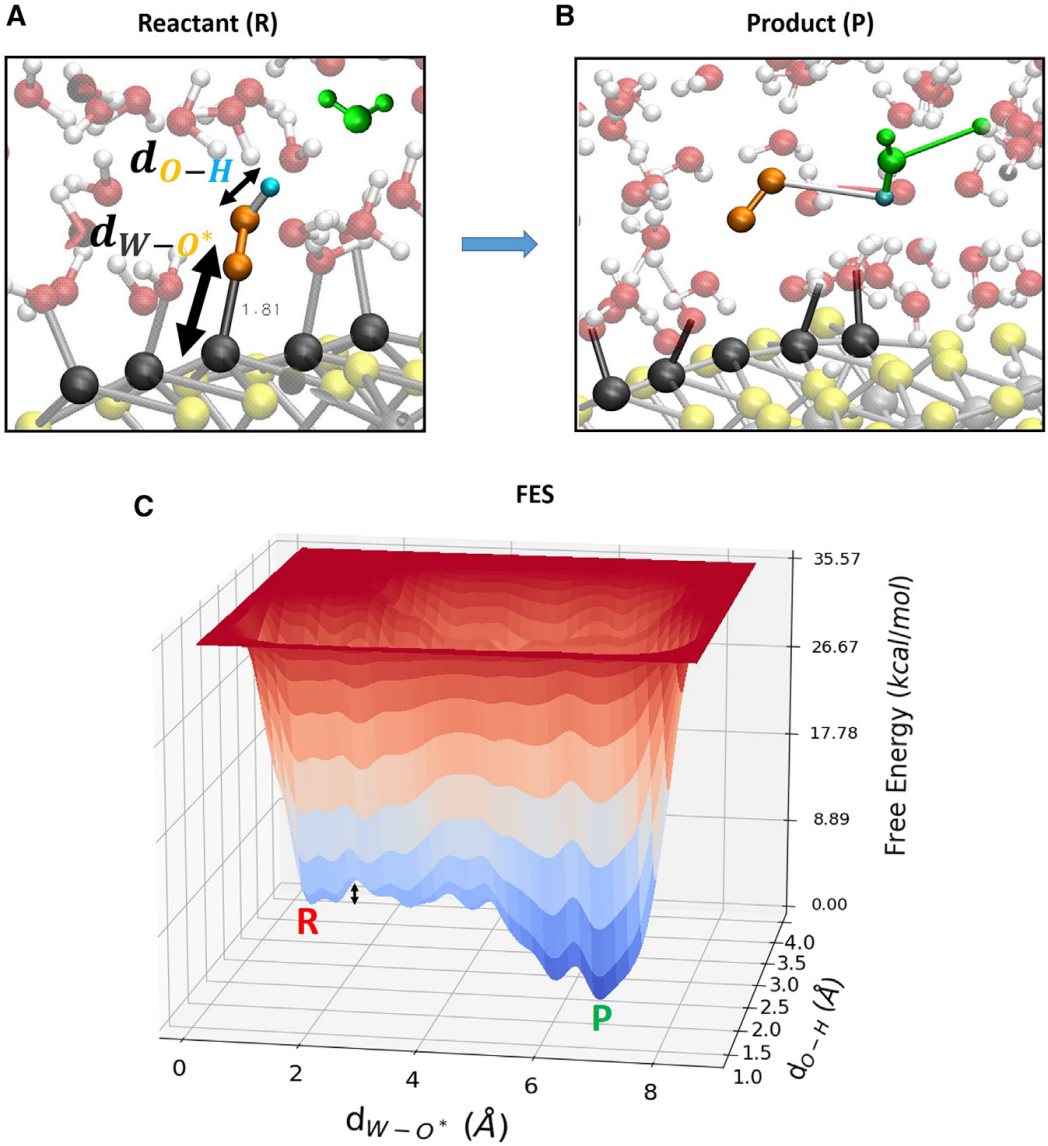
Free energy surface Snapshots from (DFT-MD) WT metadynamics simulations of reactant (A) and product (B) states for the liquid-phase OER step 4 → 5 at the (100)-WSe_2_ facet. Black arrows highlight chosen reaction coordinates. (C) The associated 3D free-energy surface. R and P denote the reactant and product state, respectively. The free-energy scale in the z axis is in kcal/mol. Reaction coordinates are expressed as a distance (Å) in x- and y axis. The black arrow highlights the free-energy barrier.

The dissociation of the reactant water molecule and consequently the formation of surface adsorbed intermediates $HOO^{*}$ (formation of $\mu_{1}$-OOH surface termination). Noticeable is that the dissociated proton from the water molecule (H^+^ is in green color in Figures 9A and 9B) is not anymore surface adsorbed such as in the previous identified gas-phase pathway, but the dissociated H^+^ hops toward a nearby water molecule belonging to the explicit liquid water environment (through a simultaneous H_3_O^+^ formation/proton transfer). The free-energy barrier is ∼ 11 kcal/mol (∼0.5 eV), slightly lower than ∼ 15 kcal/mol found in gas-phase for the same reaction step. Reaction step 4 → 5 ($HOO^{*}\to O_{2}\left(g\right)+H^{+}+e^{-}$): the free energy surface is in Figure 10C (deprotonation of $HOO^{*}$ and the subsequent $OO^{*}$ desorption from the (100)-WSe_2_ surface).

**Figure 11:**
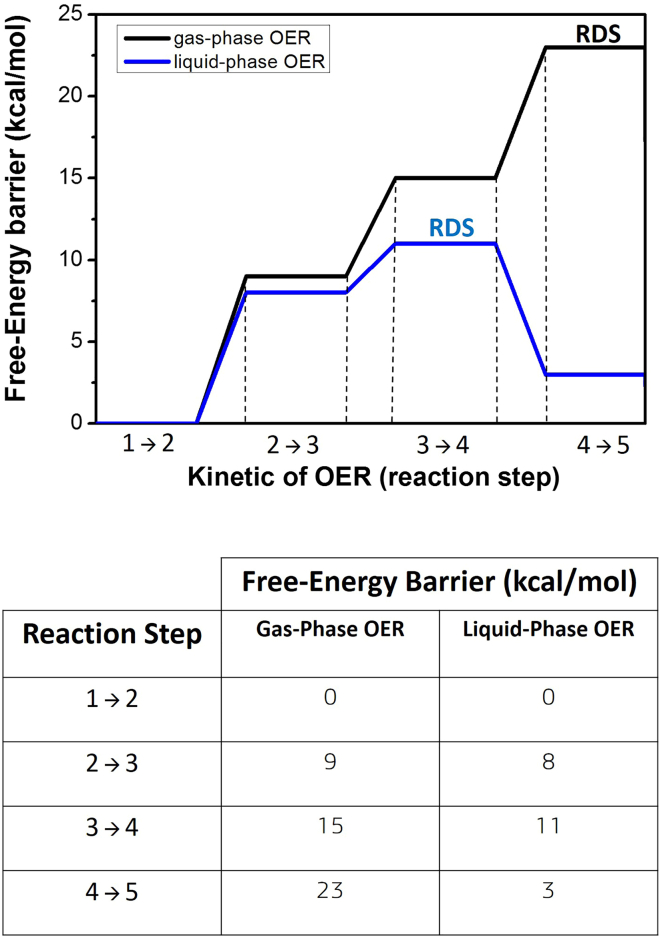
Free-energy barriers for each OER step investigated at gas and liquid-phase RDS in the graph highlights the rate-determining step.

Again, the dissociated proton (H^+^ in cyan color in Figures 10A and 10B) is not anymore surface adsorbed by a neighboring Se atom (as already seen for the previous pathway in gas-phase), but the dissociated H^+^ hops toward a nearby water molecule (belonging to the surrounding liquid water environment, in green color in Figures 10A and 10B) and, at the same time, a proton from this latter hops toward another nearby water molecule (through a simultaneous H_3_O^+^ formation/proton hop). The free-energy barrier is ∼ 3 kcal/mol (∼0.1 eV), quite lower than ∼ 23 kcal/mol found in gas-phase for the same reaction step. In agreement with our previous OER free-energy barrier investigation at the (110)-RuO_2_ ^30^ with an explicit water environment at the interface, the reaction step with the largest free-energy barrier is also here identified in the reaction step 3 → 4 ($HOO^{*}$ adsorbate), with a free-energy barrier of ∼ 11 kcal/mol (∼0.5 eV). The concerted reaction step 3 → 4 is therefore identified as the RDS of the liquid-phase OER at the (100)-WSe_2_ facet. Our results identify, within the assumptions applied in this work, two different RDS, that is the reaction step 4 → 5 ($OO^{*}$ desorption) and the reaction step 3 → 4 ($HOO^{*}$ adsorbate) for the gas- and liquid-phase OER, respectively, with different value of the associated free-energy barrier as ∼ 23 kcal/mol (gas-phase) and ∼ 11 kcal/mol (liquid-phase). Liquid-phase OER TSs are displayed in Figures S3 in the supplemental information. Note that all the free-energy barriers estimated for the OER in liquid-phase (explicit bulk water environment), are always lower than those calculated in gas-phase (see Figure 11). For the sake of clarity, our results about free-energy barriers are compared and listed in Figure 11.

The reaction in the work presented here now proceeds preferentially through a ‘water-assisted mechanism’, where explicit water molecules assist and enhance the OER to occur. More specifically, the dissociated hydrogen atoms of the reactant water molecule energetically prefer to hop toward nearby water molecules belonging to the explicit surrounding water environment instead to be surface adsorbed by Se atoms (as it happens in gas-phase, i.e., without the explicit environment). It seems that the presence of an explicit aqueous environment and accordingly the dynamics of the water environment somehow enhance the surface activity at the hydrated (100)-WSe_2_ facet: the explicit water environment does not act only as a ‘spectator’ but it plays a crucial role in the kinetic evolution of the OER participating in the reaction mechanism. Similar water-assisted mechanisms and conclusions about OER free-energies have been found in our previous OER metadynamics investigations on cobalt and ruthenium oxides.^28^^,^^30^ Noticeably is that such a water-assisted OER mechanism is different from the OER pathways proposed by Norskov et al.^37^^,^^38^ and different from most of the OER studies present in the literature (about surfaces) which are static DFT calculations and suffer from the lack of an explicit solvent, and for which the dissociated hydrogen atoms of the water molecule are therefore systematically assumed to be surface adsorbed as it happens in our gas-phase scenario in Figures 6B and 7B. However, our findings show that dissociated hydrogen atoms could energetically prefer to hop toward nearby water molecules when an explicit water environment is taken into account in DFT-MD-based calculations. The water environment and its dynamics as co-reactant strongly affect the OER minimum energy pathway showing a free energy barrier request lower than the gas-phase counterpart. Moreover, the surface catalyst sites are not anymore 3 adjacent surface sites (as detected for gas-phase OER pathway) but just adjacent $\mu_{1}$-O (W-O) and $\mu_{1}$-OH (W-OH) (and water molecules from the water environment) are enough in catalyzing the OER, as outlined in Figure 10. In a nutshell, the catalytic action of the water environment at the interface and its dynamics plays a key role in lowering the OER free-energy barrier and in identifying the minimum energy pathway and its RDS. This result is in agreement with previous evidences for which condensed-phase reactions are, in general, less demanding in terms of free energy.^89^^,^^90^^,^^91^

## Discussion

In this paper, DFT-MD simulations coupled with state-of-the-art metadynamics techniques have been applied to gain a full comprehension of the (100)-WSe_2_ aqueous interface in catalyzing the OER not only in gas-phase but also in liquid-phase at room temperature. Elucidating the detailed reaction mechanisms of water oxidation on (100)-WSe_2_ facet –as well as related free energies and surface chemical reactivity– has a crucial role in improving the efficiency of the catalyst and thus helps for a better design. Most of the theoretical and experimental attempts to understand the factors affecting the kinetics of the electrochemical water oxidation focus only on the properties of the catalysts. Those research efforts concentrate on developing/improving catalysts able to reduce the intrinsic overpotential needed at the anode for the OER occurring. Less common are theoretical research efforts to understand the role of an explicit water environment in the water (photo)electrocatalysis, which can however strongly affect the OER activity. In this paper, a study of the OER has been presented not only by looking at the WSe_2_ catalyst, but also by addressing the role of the water environment in the catalytic process. Accordingly, both gas-phase and liquid-phase OER have been investigated with the aim of revealing the associated free-energy barriers. Our findings shed light on two different reaction pathways in gas- and liquid-phase OER, described by two different RDS and related free-energy barriers. $OO^{*}$ desorption and $HOO^{*}$ adsorbate formation have been identified as the RDS in gas- and liquid-phase, respectively, with a free-energy barrier in liquid-phase that is lower than the one estimated in gas-phase. Thanks to metadynamics investigations, we step-by-step revealed the mechanism and the dynamics behind selected reaction steps of the OER, remarking a pathway characterized by water as co-reactant and co-catalyst in a sort of water-assisted mechanism able to enhance the kinetics of the OER. This dual water behavior is crucial for efficient catalysis, as it has already been suggested in our previous OER investigations on cobalt and ruthenium oxides.^28^^,^^30^ Our results support the view that the bilateral interplay between the surface catalyst and a water/solvent environment, combined with dynamic behavior at the interface are critical and not-negligible groundwork, together with free-energy barriers estimations, for a rational comprehension and design of improved catalysts in the context of electrocatalyzed water splitting reaction.

### Limitations of the study

There are still open questions to address, such as the understanding of the OER in explicit acidic or alkaline environments (for instance, by tuning surface pH and/or adding ions at the interface). OER DFT-based investigations with a proper inclusion of electrochemical conditions^92^ is also a subject to address; additionally, studying how temperature variations impact reaction mechanisms and catalyst’s stability could be essential. Reducing the computational cost of dynamic simulations (like DFT-metadynamics) is an additional research area, where machine learning and data-driven approaches have become increasingly common for predicting catalytic activity and optimizing catalysts at lower computational costs.^71^^,^^72^^,^^73^^,^^74^^,^^75^^,^^76^ However, further research is needed to advance these methods in this field.

## Resource availability

### Lead contact

Further information and requests for resources should be directed to and will be fulfilled by the lead contact, Fabrizio Creazzo (fabrizio.creazzo@chem.uzh.ch).

### Materials availability

This study did not generate new unique reagents.

### Data and code availability

• Data: *xyz* coordinates of all our reactant and product states in gas phase and in liquid phase can be found in the Mendeley data repository (Creazzo, Fabrizio (2024), *Data S2 XYZ*, Mendeley Data, V1, https://doi.org/10.17632/vx2g6p7w3j.1).
• Code: details in the key resources table*.*
• Other: see the STAR Methods section for method details. Additional data only used for the publication of this manuscript are available from the lead contact on request.

## Acknowledgments

This work is embedded as part of the National Centers of Competence in Research-Catalysis (NCCR-Catalysis) Swiss National Science consortium (Grant No 1-006445-074) and the Swiss National Science Spark grant (Grant No CRSK-2-220781). We acknowledge PRACE for awarding us access to Piz Daint, at the Swiss National Supercomputing Center (CSCS), Switzerland (project ID: pr119).t.

## Author contributions

F.C. primarily performed DFT-MD/metadynamics simulations, analyzed data, performed statistical analysis, validated results, and wrote/edited the manuscript. K.S. co-supervised the work, provided experimental information, and discussion and edited the manuscript. S.L. supervised the work, checked computational results, edited the manuscript, and obtained the funding.

## Declaration of interests

The authors declare no competing interests.

## STAR★Methods

### Key resources table

**Table:** 

REAGENT or RESOURCE	SOURCE	IDENTIFIER
**Software and algorithms**		

CP2K	CP2K^93^^,^^94^	https://www.cp2k.org/
PLUMED	PLUMED^95^	https://www.plumed.org//
Data S2 XYZ	Mendeley data repository	https://data.mendeley.com/datasets/vx2g6p7w3j/1

### Method details

**Figure 12:**
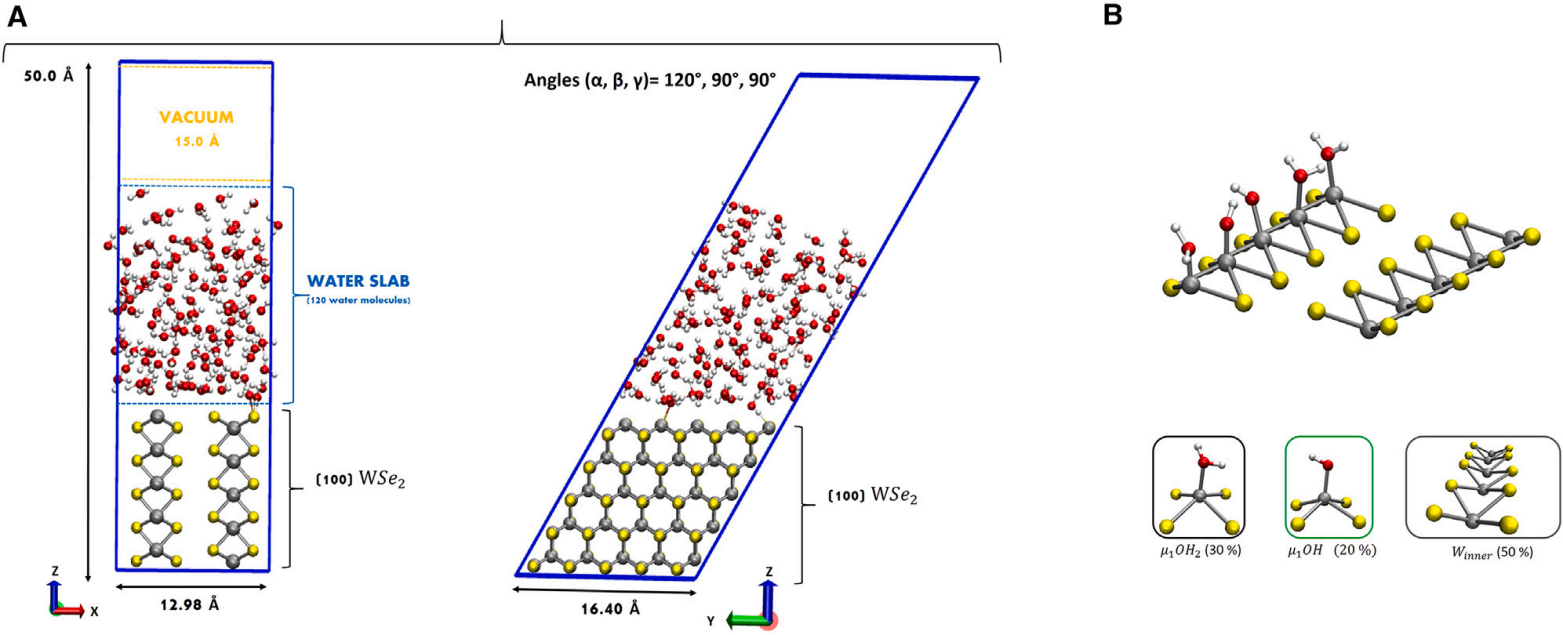
DFT-Modeling (A) Simulation box for the DFT-MD of (100)-WSe_2_-liquid water interface 510 atoms: 150 solid atoms, 120 water molecules (360 atoms). Only one surface (the upper) is put in contact with liquid water. The bottom surface is in contact with the vacuum. (B) Equilibrium composition and speciation of the (100)-WSe_2_ surface in contact with liquid water. Tungsten, oxygen and hydrogen atoms are colored in gray, red and white color, respectively. Reprinted (adapted) with permission from ref.^31^

We adopted a computational setup that has been tested in our preliminary investigation on the modeling of WSe_2_.^30^ In a nutshell, spin-polarized Kohn-Sham DFT-MD simulations (Born-Oppenheimer framework) have been performed via the CP2K program package.^93^^,^^94^ Perdew-Burke-Ernzerhof (PBE)^96^ and GTH^97^ are adopted as exchange-correlation functional and pseudopotential, respectively, which have been used previously for the description of properties of WSe_2_ and liquid water.^98^^,^^99^^,^^100^^,^^101^ The DZVP-MOLOPT-SR basis set^102^ and a 400 Ry plane-wave basis set have been used, and dispersion interactions have been taken into account by Grimme’s D3 correction.^103^^,^^104^ Periodic boundary conditions (PBCs) have been applied in all three spatial directions. The spin-polarized DFT-MD simulation has been performed on (100)-WSe_2_/liquid water interface in the NVT ensemble (∼ 25 ps), where the temperature was kept constant at 300 K by a Nosé-Hoover chain thermostat.^105^ The Velocity-Verlet algorithm^106^ with a time step of 0.5 fs has been used. Total spin-multiplicity default value (=0) has been set. Calculations have been performed with a constant total number of electrons (constant charge) as in many theoretical previous studies, see e.g Refs.^37^^,^^38^^,^^39^^,^^107^ The PBE functional was supplemented with the Hubbard U parameter^108^^,^^109^ in order to circumvent overdelocalization errors in the exchange and correlation of *d*-orbitals (and the consequent underestimation of the band gap).^110^ The Hubbard parameter U is known as the on-site Coulomb interaction energy.^111^ The optimum U parameter values have been calculated in our previous paper^31^ for the WSe_2_ bulk structure, namely U=5 eV, U=4 eV for $W^{4+}$ and Se^2–^ respectively, confirming WSe_2_ to be a semiconductor with the expected electronic band-gap. Details about calculations of the U parameter for the WSe_2_ can be found in our previous paper.^31^ The (100)-WSe_2_ at the interface with water has been modelled in our previous paper^31^ as a 5 layers slab (symmetric and stoichiometric) of 150 atoms in contact with a bulk liquid water environment, and a vacuum of 15.0 Å along the z-direction above the liquid has been included to separate the periodic *z*-replicas. The simulation box and its dimensions are shown in below Figure 12A.

In our previous work, ref.^31^, we explored water adsorption mechanisms, water organization and WSe_2_ bulk model properties. Adsorption of water molecules (entire or dissociated) at the (100) surface have been observed.^31^ Such a hydrated (100)-WSe_2_ facet exposes therefore several surface chemical species, listed in Figure 12B, as a result of (100) surface wettability/hydroxylation: only 5 water molecules (3 entire and 2 dissociated) are surface-adsorbed at the unsaturated $W_{cus}$ leading to a surface termination/speciation with 30% of $\mu_{1}$-OH_2_ and 20% of $\mu_{1}$-OH exposed sites over $W_{cus}$ atoms, as depicted in Figure 12B. $W_{inner}$ sites are not affected by the presence of water at the surface (see Figure 12B). The unsaturated Se_2_ atoms at the (100) surface receive the two hydrogen atoms (from the two dissociated water molecules at $W_{cus}$ sites) leading to two Se-H exposed sites and keeping the surface pH (=7) neutral. We found that the hydrated (100) surface can be defined as a hydrophobic surface where a preferential in-plane organization of interfacial H-bonded water molecules (2D-HB-network^112^^,^^113^^,^^114^) has been detected. Moreover, we calculated the electric field and related work function of the (100)-WSe_2_ surface at the interface with vacuum and with the water slab. We found an electric field of ∼ 6.5 V/ Å at the (100)-WSe_2_/liquid water interfacial system, and a surface work function of ∼ 2.3 eV. See ref.^31^ for further details.

#### Metadynamics

**Figure 13:**
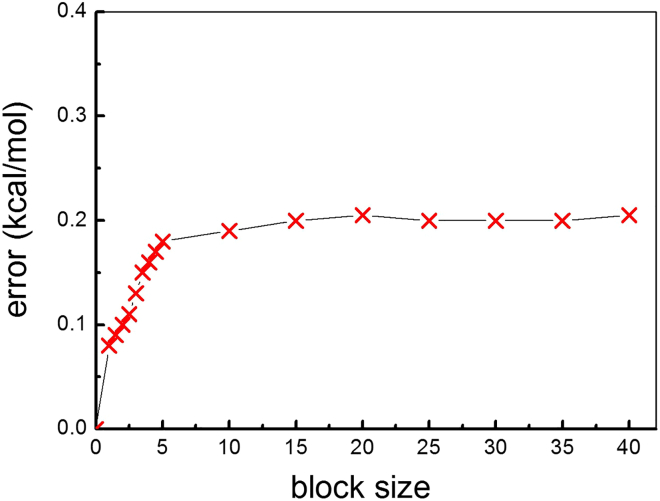
The error of FESs obtained from block analyses

The PLUMED-2.7 software package,^95^ within the CP2K code,^93^^,^^94^ has been used for the free-energy landscape reconstruction on gas-phase and liquid-phase OER at (100)-WSe_2_ facet. Enhanced sampling techniques in the DFT-MD well-tempered (WT)^58^ metadynamics framework have been adopted. WT metadynamics has been shown to be a successful enhanced sampling method for the investigation of (multi-step) reactions,^51^^,^^52^^,^^115^^,^^116^ such as in our case. The free-energy profiles have been obtained by exploring the (local) configurational space (*i.e.*, the phase space) and hence probing the relevant (meta)-stability basins and the connecting chemical pathway on the space spanned by the chosen collective variables (CVs, or reaction coordinates). The modeling of the OER and adopted CVs for our metdaynamics investigations are detailed in section *Our modeling of the OER at the (100)-W*Se_2_. Transition states (TSs) displayed in Figures S2 and S3 in the supplemental information were identified by following the minimum energy pathway (MEP) on the free energy surfaces. The MEP is the trajectory in the multidimensional free energy surface connecting reactants and products, while passing through the saddle point (the TS), by minimizing the energy barrier. Each reaction step, in both gas and liquid phase, has been investigated with an average simulation time of at least 30 ps, checking and assuring a proper sampling convergence by monitoring the Gaussians addition workflow as a function of the simulation time and by estimating an error of 0.2 kcal/mol via block analyses averaging and reweighting on more CVs^117^ (see below Figure 13), giving reliability to the the phase-space spanning in question. 1.2 kcal/mol (initially) and 0.02 Å are respectively the height (*W*) and the width ($\sigma_{s}$) of the Gaussian hills added along the WT biased metadynamics trajectories, as adopted in our earlier WT metadynamics investigation.^30^ See also Figure S1 in the supplemental information about distances between reactant atoms over simulation time (ps) for each reaction step investigated in gas-phase and liquid phase by DFT-metadynamics. A (maximum) upper-wall distance of 5 Å has been used for the phase-space sampling in liquid-phase OER investigations. The upper-wall distance has been increased up to 8 Å for gas-phase OER investigations. A longer upper-wall distance was needed for a proper phase-space sampling and calculation convergence, due to a wider range of configurations that the reactant water molecule in gas-phase, less spatially and geometrically constrained than in liquid-phase, could explore in the free energy landscape. The initial Gaussian potential height was automatically reduced during the exploration of the configurational space as the filling procedure progressed in time, as a standard procedure in WT metadynamics, accordingly to a bias-factor *γ* of the WT-metadynamics set to 4.^58^ A Gaussian hill of decreasing *W* value was added every 50 fs (pace of 100 steps), and no other external biases (such as external potential) or pH changes have been adopted.

### Quantification and statistical analysis

CP2K program package^93^^,^^94^ has been used to perform DFT-based calculations. PLUMED-2.7 software package,^95^ within the CP2K code,^93^^,^^94^ has been used to perform well-tempered (WT)^58^ metadynamics.
