# Supplementary material for: DFT-metadynamics insights on the origin of the oxygen evolution kinetics at the (100)-WSe2 surface
Source: iScience. 2025 Feb 20;28(3):112045. doi: 10.1016/j.isci.2025.112045 (PMC11930373; doi:10.1016/j.isci.2025.112045)
Supplement: Document S1. Figures S1–S3 [file mmc1.pdf]

iScience, Volume *28*

Supplemental information

DFT-metadynamics insights on the origin of the oxygen evolution kinetics at the (100)-WSe_2 surface

Fabrizio Creazzo, Kevin Sivula, and Sandra Luber

S1. CV AS A FUNCTION OF DFT-METADYNAMICS SIMULATION TIME

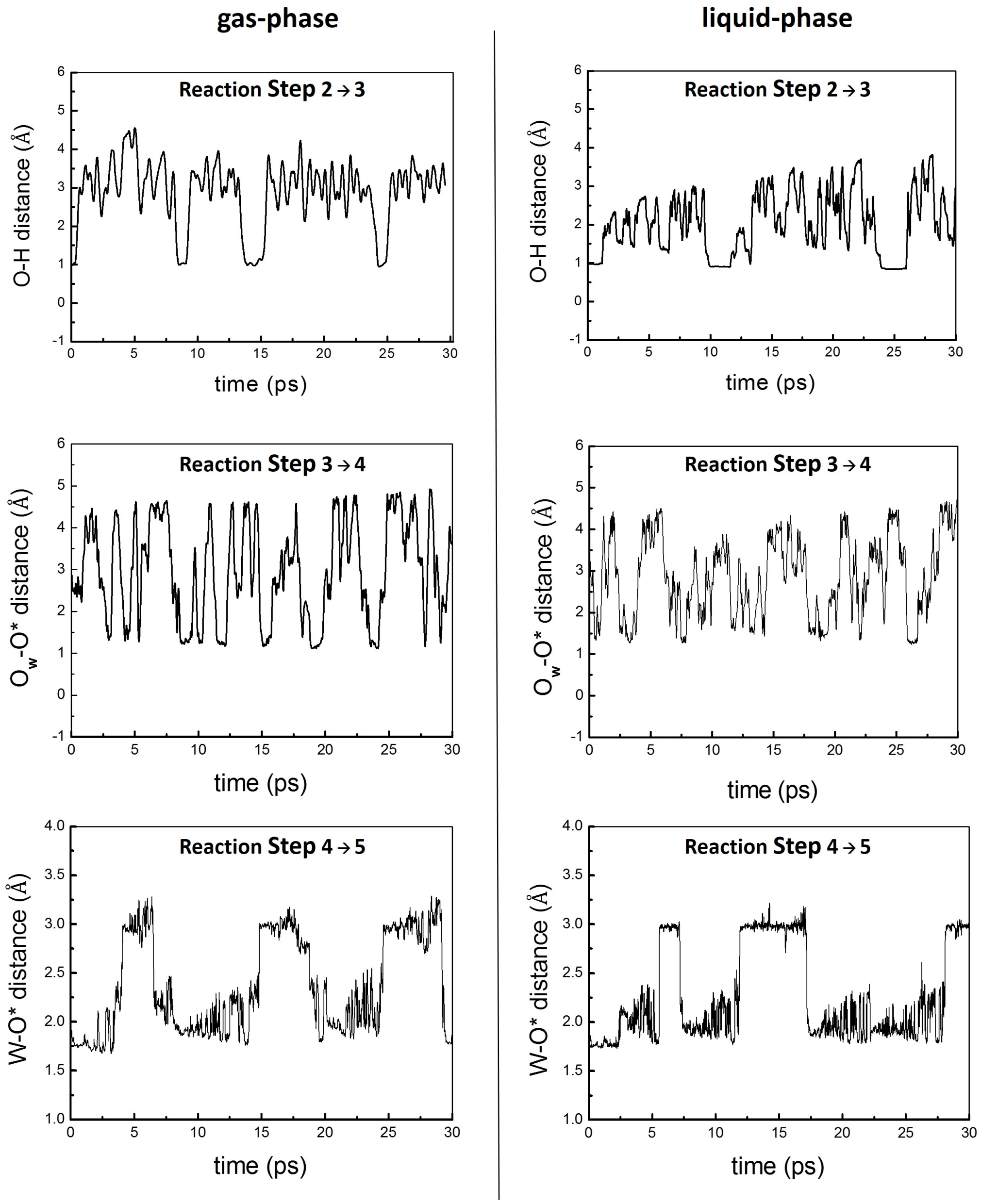

FIG. S1. *Distance (Å) between reactant atoms over simulation time (ps) for each reaction step investigated in gas-phase and liquid phase by DFT-metadynamics. The x-axis represents the simulation time in ps, and the y-axis represents the distance in Å. The distances oscillate between characteristic values corresponding to the reactant and product states, revealing multiple transitions between reactant and product. This latter is a hallmark of the method's ability to explore and fill the free energy landscape effectively.*

S2. OER REACTION PATHWAY IN GAS PHASE

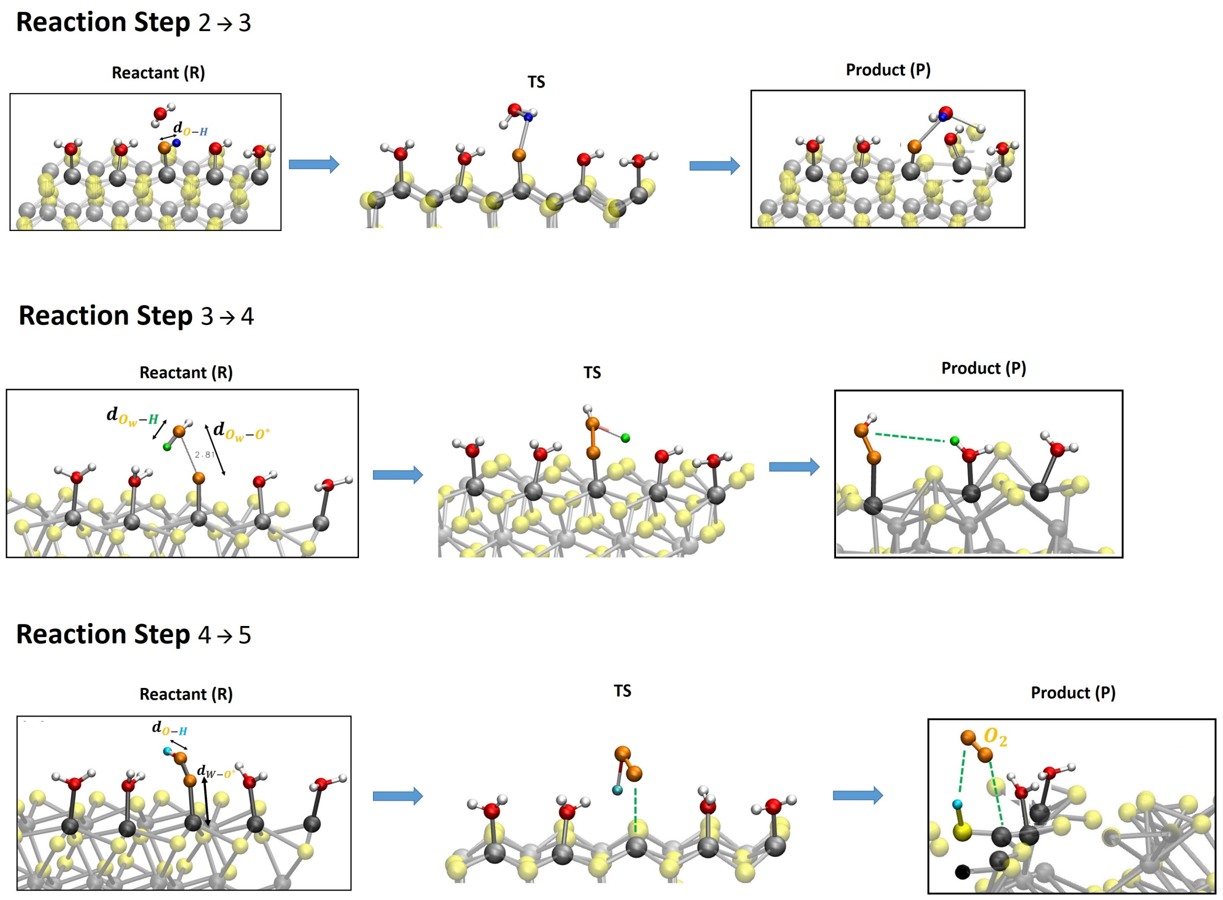

FIG. S2. *Reaction pathway of each reaction step of the OER. TS is the transition state identified by following the minimum energy pathway on the free energy surface. The black arrow highlights the chosen reaction coordinate for DFT-metadynamics investigations.*

S3. OER REACTION PATHWAY IN LIQUID PHASE

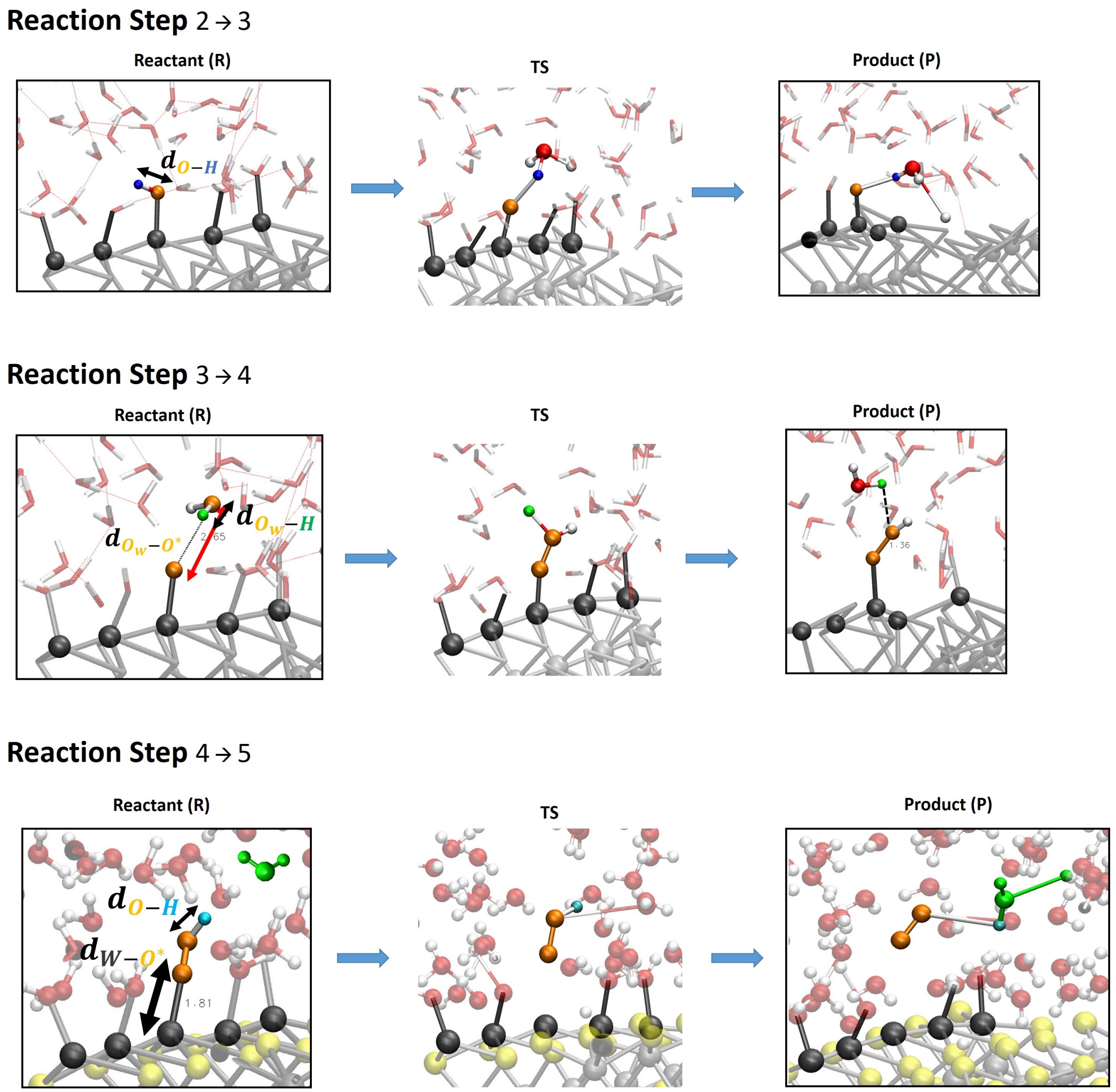

FIG. S3. *Reaction pathway of each reaction step of the OER. TS is the transition state identified by following the minimum energy pathway on the free energy surface. The black arrow highlights the chosen reaction coordinate for DFT-metadynamics investigations.*
